# Thermally drawn multi-material fibers: from fundamental research to industrial applications

**DOI:** 10.1093/nsr/nwae290

**Published:** 2024-08-24

**Authors:** Xin Chen, Yan Meng, Stella Laperrousaz, Hritwick Banerjee, Jinwon Song, Fabien Sorin

**Affiliations:** Institute of Materials, École Polytechnique Fédérale de Lausanne, Switzerland; Institute of Materials, École Polytechnique Fédérale de Lausanne, Switzerland; Institute of Materials, École Polytechnique Fédérale de Lausanne, Switzerland; Institute of Materials, École Polytechnique Fédérale de Lausanne, Switzerland; Institute of Materials, École Polytechnique Fédérale de Lausanne, Switzerland; Institute of Materials, École Polytechnique Fédérale de Lausanne, Switzerland

## Abstract

Thermally drawn fiber devices, with their complex micro- to nanoscale architectures, hold great promises not only for scientific research but also for scalable industrial applications in soft smart systems.

Amidst technological advancements in intelligent sensors and e-textiles, many challenges are faced when combining robustness with distributed and precise multimodal monitoring in traditional node sensors. Thermally drawn fiber devices, which exhibit extended length and embed multi-material architectures with complex geometries at the micro- to nanoscale, can constitute alternative candidates for soft and large-scale smart systems [1]. The thermal-drawing process involves heating a macroscopic preform to above materials-softening temperatures (the glass-transition temperature *T_g_* for glasses or the melting temperature for crystalline constituents) and stretching the preform into kilometer-long fibers (Fig. 1a and b). To ensure uniform pulling, the fiber is attached to a rotating capstan set at a controlled speed (*v_draw_*), while the preform is fed into the furnace at speed (*v_feed_*). Volume conservation imposes a relationship between the diameter of the preform (*D_preform_*) and the diameter of the fiber (*D_fiber_*) that brings out the scaling effect of all the cross-sectional dimensions that shrink down to the micro- or nanoscale, while the fiber length increases accordingly:


$${{D}_{{fiber}}} = {{D}_{{preform}}}\sqrt {\frac{{{{v}_{{feed}}}}}{{{{v}_{{draw}}}}}}.$$

**Figure 1.**
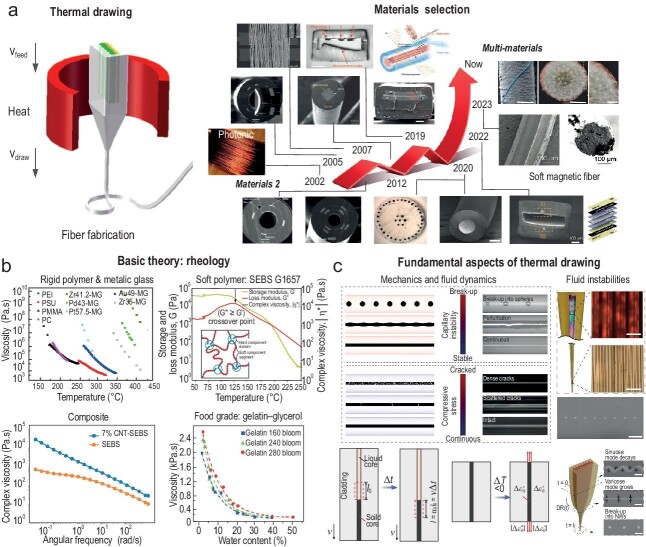
(a and b) Basic theory and development of materials available for thermal drawing. Reproduced with permission [1,17–20]. (c) The integration of fundamental theory with the thermal-drawing technique [8,12].

The field of thermally drawn multi-material fibers that integrate materials beyond the traditional glasses or thermoplastics used for optical fibers was born ∼20 years ago with the demonstration of the first metal, semiconductor and insulator optoelectronic fiber [2,3]. This breakthrough has unlocked several research thrusts to integrate novel materials that are compatible with this process, including crystalline and amorphous metals, semiconductors, piezoelectrics, elastomers, biodegradable and even food-grade materials (Fig. 1a). This trend for integrating novel materials, as well as increasingly complex architectures and functionalities within thin and sometimes soft fiber, is growing, with many active research groups in top-ranking universities across the world in this field.

The first phase of the multi-material fiber field was focused on the demonstration of several proof-of-concepts fiber devices based on novel materials and fiber configurations. It included, to a limited extent only, an in-depth investigation of the fundamental aspects regarding materials and processing associated with thermal drawing. Breakthroughs in fundamental science are of paramount importance to support the development of novel technologies towards differentiating products with societal and economical impacts. In the last decade, publications in the field have seen an increase in studies looking into deeper scientific aspects. For example, in-depth rheological and microstructural understanding has unlocked the ability to fabricate ultra-stretchable fiber devices, as well as biodegradable and food-grade polymers [4,5]. A combination of similar rheological study, combined with fluid-dynamics modeling and advanced nano-metallurgy analysis, has enabled the thermal drawing of metallic glasses [6]. The advancement of fluid-dynamics modeling is also key to a better understanding of the fundamental aspects behind the drawing of nanocomposites with micro- and nanoscopic textures, as well as the formation of filaments or droplets during and after drawing [7–12]. Other very recent breakthroughs (Fig. 1c) have tackled the understanding of cracks and deformations of semiconducting cores during fiber drawing induced by stress and capillary instabilities. Supported by advanced mechanical and hydrodynamic modeling, a new cladding material was proposed that reconciles the requirements of the drawing temperature, thermal expansion and viscosity, realizing long and robust semiconductor fibers, opening up great opportunities in flexible and wearable electronics [8]. In the same spirit, by combining fluid dynamic analysis of the thermal-drawing process coupled with engineered capillary instabilities, a scalable method to produce encapsulated flexible nanowire (NW) arrays was recently proposed (Fig. 1c) [12]. Regarding smaller scales, investigation of the microstructure of post-drawing polymers using advanced X-ray scattering analysis has revealed the effect of stress on chain alignment. Such fundamental understanding paves the way towards exploiting similar principles to realize artificial muscles based on advanced fibers [13]. Finally, novel approaches to functionalize thermally drawn fibers have emerged with exciting new opportunities, particularly in bioengineering [14,15].

Through this succinct account of recent work looking at fundamental breakthroughs in the materials and processing aspects of multi-material fibers, it is clear that this field has developed a strong scientific basis on which to grow. For a scientific and technological field to keep momentum and thrive, however, it is very important that the fundamental science-enabled breakthroughs find practical applications with a significant economic or societal impact. This keeps the various stakeholders engaged, maintains the interest of funding agencies and increases the volume of funding. It also sustains interest from academic institutions and promotes more collaboration with industrial partners. The next pivotal development phase in the field should hence turn its focus also on successfully transitioning laboratory demonstrations into industrial products that can positively impact society.

Multi-material fibers offer great potential in fields such as neural probes, soft robotics, sensing, e-textiles, wearables for health monitoring, human–machine interaction or bio-implantation. A few examples include thermally drawn polymer-based fibers with novel materials for recording and stimulation, as well as microelectronic chips for data transfer [16–18] (Fig. 2a). With the ability to integrate micro-channels, optical fibers and electrodes, flexible multi-material fibers can constitute a great asset for medical or sensing probes [19,20]. Moreover, fibers made out of elastomers open a breadth of novel opportunities for soft electronics, soft robotics and diagnostic and surgical tools, as well as for sensors and actuators [21–24].

**Figure 2.**
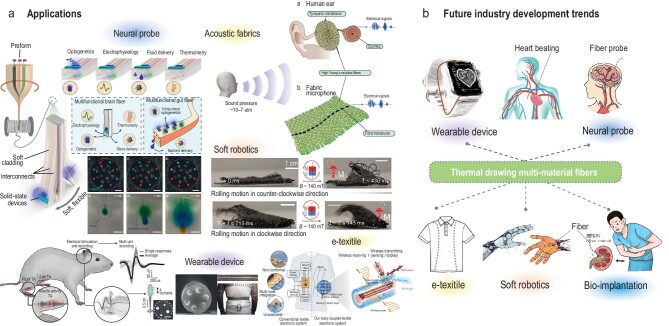
(a) Preliminary attempts at thermally drawn fibers in various application domains. Reproduced with permission [1,8,10–13,17,21,23,24]. (b) Potential markets for thermally drawn fibers.

To ease adoption of these materials, their durability, sensitivity, miniaturization and bio-compatibility need to improve (Fig. 2a and b). Moreover, while optical fiber connectors have developed tremendously, the more recent need for fiber electrical connection will require innovative approaches to interface efficiently and in a robust industrial way with the different conducting materials embedded within advanced fibers. Here, the versatility of the fiber architectures can be put to advantage to design fibers with specific configurations that can ease fiber connection. The advancement of additive manufacturing and digitization can also have a significant impact on facilitating the scaling-up of the preform fabrication stage and post-drawing fiber assembly, while complicating the resulting fiber architectures [25]. The development of Artificial Intelligence-supported manufacturing and device designs can also constitute a novel leverage for the field of multi-materials fibers, thus far seldomly explored.

Several platforms that are going in this direction are now emerging to allow a transition from fundamental science to proof of concept and towards fiber products. The advanced Fibers and Fabrics of America is a leader in this effort, together with newly established ventures such as FiberLab in Switzerland and NeuroBionics in the USA. Direct industrial partnerships and ventures also constitute an important leverage to bring fiber technologies into the market, with encouraging emerging projects on the horizon [26,27]. These developments are crucial in order for the field to continue to thrive and leverage the unique value proposition of multi-material fibers to positively impact science and technology, as well as the economical and societal landscapes.
